# Epicardial Ablation for Arrhythmogenic Disorders in Patients with Brugada Syndrome

**DOI:** 10.3390/biomedicines13010027

**Published:** 2024-12-26

**Authors:** Andrea Matteucci, Marco Valerio Mariani, Luca Sgarra, Michela Bonanni, Marco Frazzetto, Vincenzo Mirco La Fazia, Nicola Pierucci, Carlo Lavalle, Claudio Pandozi, Federico Nardi, Furio Colivicchi

**Affiliations:** 1 Clinical and Rehabilitation Cardiology Division, San Filippo Neri Hospital, 00135 Rome, Italy; 2 Department of Experimental Medicine, Tor Vergata University, 00133 Rome, Italy; 3 Department of Cardiovascular, Respiratory, Nephrological, Aenesthesiological and Geriatric Sciences “Sapienza” University of Rome, 00185 Rome, Italy; 4 Cardiology Department, Regional General Hospital “F. Miulli”, 70021 Bari, Italy; 5 Fondazione Toscana G. Monasterio, Ospedale del Cuore, 54100 Massa, Italy; 6 Division of Cardiology, Harrington Heart & Vascular Institute, University Hospitals Cleveland Medical Center, Cleveland, OH 44106, USA; 7 Texas Cardiac Arrhythmia Institute, St. David’s Medical Center, Austin, TX 78705, USA; 8 Santo Spirito Hospital, Casale Monferrato, 15033 Alessandria, Italy

**Keywords:** Brugada syndrome, epicardial ablation, sudden cardiac death

## Abstract

Brugada syndrome (BrS) is an inherited arrhythmogenic disorder characterized by distinct electrocardiographic patterns and an increased risk of sudden cardiac death due to ventricular arrhythmias. Effective management of BrS is essential, particularly for high-risk patients with recurrent arrhythmias. While implantable cardioverter–defibrillator (ICD) is effective in terminating life-threatening arrhythmias, it does not prevent arrhythmia onset and can lead to complications such as inappropriate shocks. Epicardial ablation has emerged as a promising treatment option for patients with recurrent ventricular arrhythmias and frequent ICD interventions. This review examines the latest advancements in the management of Brugada syndrome, focusing on the role and rationale of epicardial ablation for the treatment of patients at risk of sudden cardiac death.

## 1. Characteristics of Brugada Syndrome

Sudden cardiac death (SCD) is a dominant cause of outcome, especially in older populations due to ischemic heart disease, or in healthy individuals without apparent structural heart disease experience episodes of ventricular life-threatening arrhythmias.

**Figure 1:**
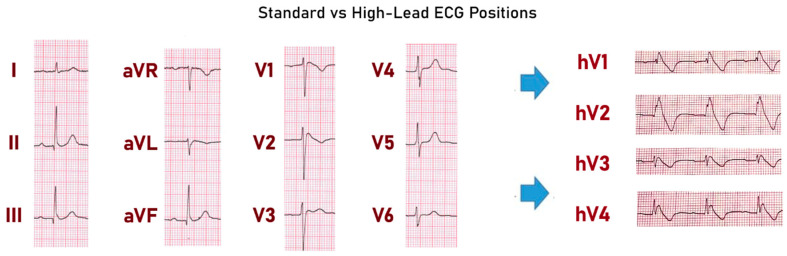
Comparison between standard chest electrode placement and high-lead placement in patients with BrS. (**Left**): Standard precordial lead placement, with V1 and V2 positioned in the fourth intercostal space. (**Right**): High-lead placement to enhance diagnostic sensitivity in Brugada Syndrome. In this configuration, V1 and V2 are shifted to the second or third intercostal space, remaining parasternal, while V3 and V4 are similarly elevated to corresponding higher positions along the chest.

Brugada syndrome (BrS) is an inherited arrhythmogenic disorder primarily affecting the epicardium of the right ventricular outflow tract (RVOT). The electrical instability seen in many electrical disorders such as in BrS, creates a pro-arrhythmic environment despite the lack of macroscopic structural abnormalities [1,2,3]. The distinctive ECG features of the Brugada pattern are a J-point elevation of at least 0.2 mV, along with a coved ST-segment elevation and T-wave inversion in one or more right precordial leads, specifically V1 or V2, positioned in the second, third, or fourth intercostal spaces (Figure 1).

This pattern can present either on its own or be triggered by sodium channel-blocking drugs or fever. BrS diagnosis is given to patients with spontaneous type 1 ECG pattern without other cardiac conditions and documented polymorphic ventricular tachycardia (VT) or ventricular fibrillation (VF), syncopal events due to arrhythmias, or relevant family history. Genetic testing in BrS reveals mutations impacting sodium, calcium, and potassium channels, with SCN5A mutations affecting the sodium channel α-subunit identified in 20–30% of cases [4]. These mutations typically result in reduced sodium current (I_Na_) and a loss of function in the SCN5A gene [5].

Implantable cardioverter–defibrillator (ICD) is reasonable in BrS patients who present symptoms and have either survived a cardiac arrest or have a record of spontaneous, sustained ventricular arrhythmias [6]. Syncope affects about one-third of BrS patients; those with unexplained episodes of fainting have a quadrupled risk of arrhythmic events compared to those without symptoms. Most individuals newly diagnosed with BrS show no symptoms, and the annual rate of arrhythmic events is around 0.5%, which complicates accurate risk assessments. A history of ventricular arrhythmias or unexplained syncope before diagnosis, male sex, and specific SCN5A mutation types characterize the fixed risk profile of each BrS patient. This inherent risk is independently linked to life-threatening arrhythmic events, irrespective of clinical symptoms or ECG pattern type [7]. The role of electrophysiological studies is still debated [8]. The primary approach for preventing SCD in BrS involves the use of an ICD, particularly in individuals with a history of cardiac arrest or syncope [9]. For patients experiencing frequent ICD shocks from VF, treatments such as quinidine or catheter ablation have proven effective in reducing these shocks. For pharmacological management, quinidine remains the only long-term therapeutic option, demonstrating effectiveness in reducing arrhythmogenic events [10]. However, treatment plans should be individualized based on patient risk profiles. For asymptomatic patients without a spontaneous type I ECG pattern, ICD is not indicated even if a drug-induced ECG reveals such changes; regular monitoring suffices in these cases. Ablating arrhythmogenic fibrotic area in the epicardial RVOT was shown to cut down on recurrent VF episodes in patients with ICD and normalize the ECG in most cases [11].

## 2. Risk Stratification

Risk stratification in Brugada Syndrome (BrS) remains a complex but essential aspect of clinical management, particularly for patients undergoing interventions such as endocardial or epicardial transcatheter ablation. Despite the availability of established risk factors such as spontaneous type-1 ECG patterns, QRS fragmentation, family history of ASCD, and syncope, SCN5A mutation subtype, these markers alone often fall short in accurately stratifying ASCD, particularly in asymptomatic individuals [7]. Only a limited number of the various tools available have been rigorously evaluated in independent cohorts with strong predictive metrics. The BRUGADA-RISK score has provided a robust framework for identifying patients at higher risk of ventricular arrhythmias or ASCD [12], identifying four independent predictors of ventricular arrhythmias/SCD: probable arrhythmia-related syncope, spontaneous type 1 Brugada ECG pattern, early repolarization in peripheral leads, and type 1 Brugada pattern in peripheral leads. These factors were incorporated into a comprehensive risk model with high predictive accuracy and specificity for risk assessment. The identified risk factors in the BRUGADA-RISK model may reflect the underlying characteristics of the substrate. For instance, spontaneous type 1 Brugada ECG pattern and early repolarization in peripheral leads suggest heightened electrical heterogeneity and a propensity for arrhythmic triggers. Patients with arrhythmia-related syncope and multiple additional risk markers demonstrated the highest ventricular arrhythmias/SCD rates during follow-up. This subset of patients, with an annual event rate exceeding 2%, represents an optimal target for proactive interventions, including epicardial ablation. Patients with a five-year predicted risk exceeding 5%, based on the BRUGADA-RISK model, may represent a population where the benefits of ablation outweigh the procedural risks. Such patients might benefit from substrate homogenization, particularly if other standard preventive measures, such as ICD, are contraindicated or less effective due to recurrent device therapies or patient preference.

The Bruxelles experience instead developed a risk score incorporating six key predictors [13]: clinical presentation, spontaneous Type I ECG, familial SCD, sinus node dysfunction (SND), programmed ventricular stimulation (PVS) inducibility, and proband status. These factors are validated through long-term follow-up and a separate external cohort affirming their generalizability. However, differences in demographic and clinical profiles between cohorts suggest the need for contextual application. For instance, spontaneous Type I ECG was associated with a hazard ratio (HR) of 2.7 for future events. Patients presenting with aborted SCD exhibit a high event rate of 11.1% per year, justifying their categorization as high-risk individuals. Syncope, particularly of suspected arrhythmic origin, also conveys substantial prognostic weight, with an HR of 3.7. In contrast, asymptomatic patients represent a more diverse cohort with variable risk profiles, complicating management decisions. Familial history, particularly early SCD in first-degree relatives, has been confirmed as a significant predictor of adverse outcomes, with a nearly threefold increase in risk (HR 2.9). This finding aligns with the genetic underpinnings of BrS, where pathogenic mutations in SCN5A and other genes contribute to arrhythmogenic vulnerability. Also, the inducibility of ventricular arrhythmias during PVS emerged as a strong independent predictor, with an HR of 4.7. Although previous literature offers conflicting views on the usefulness of PVS, their study reinforces its potential role, particularly considering that the application of less aggressive pacing protocols in their cohort further underscores the specificity of inducibility as a marker of the arrhythmic substrate. In addition, there seems to be an association of SND with increased arrhythmic risk (HR 5.0). Although SND is a relatively rare manifestation in BrS (2% prevalence), its presence often signifies a severe phenotype, particularly in younger patients [13].

## 3. Genetics Beyond Brugada Syndrome

While ablation primarily addresses arrhythmogenic substrates associated with BrS, the genetic and molecular complexities of the syndrome influence its therapeutic success and risk stratification. Beyond SCN5A mutation lies an oligogenic or polygenic condition of which we will emphasize only those to date that are most relatable for risk stratification and procedural success (Table 1).

Variants in more than 25 genes, including those encoding for ion channels and regulatory proteins, are implicated in the pathophysiology of BrS [14]. However, the identification of genetic causality remains challenging. Only 20-30% of BrS patients have SCN5A mutations, and the majority of cases remain genetically undetermined.

The presence of SCN5A mutations and their functional consequences, such as reduced I_Na_ density or altered gating properties of the Nav1.5 channel, can influence the extent and nature of the substrate. For instance, truncation variants associated with haploinsufficiency produce greater reductions in I_Na_, leading to more severe clinical phenotypes and, potentially, more extensive substrate abnormalities [15].

Moreover, genetic modifiers, such as H558R in SCN5A, can mitigate BrS severity, potentially altering the arrhythmogenic substrate [16]. Variants in genes like SCN10A, CACNA1C, and others influencing transient outward potassium current (K_Ito_)or L-type calcium channel (I_CaL_) currents may also indirectly affect substrate characteristics. Patients with gain-of-function variants in KCND3 or KCNE3, for example, experience increased K_Ito_, contributing to phase-1 action potential repolarization and arrhythmogenesis [17,18]. Ablation may provide symptomatic relief and reduce arrhythmia recurrence by modifying electrical substrates. However, it does not address the underlying genetic predisposition, leaving patients susceptible to other manifestations of the disease, such as atrial fibrillation or conduction disorders. Functional studies have demonstrated that SCN5A-related phenotypes can overlap significantly with long QT syndrome (LQTS), conduction disorders, and structural defects [14,19]. Recent studies have identified potential roles for the presence of copy number variations in SCN5A and emerging genetic modifiers, such as ZFHX3 and HEY2, that may affect conduction properties or ion channel regulation [16,20]. Additionally, emerging evidence suggests that factors beyond ion channel dysfunction, such as structural changes, fibrosis, or cholesterol metabolism, may contribute to BrS substrate formation [21,22,23]. The potential for tailored therapeutic approaches, combining genetic screening with advanced ablation techniques, holds promise for improving outcomes in BrS patients. However, further research is needed to translate genetic discoveries into actionable clinical pathways.

## 4. Pathophysiology Supporting Epicardial Ablation

**Figure 2:**
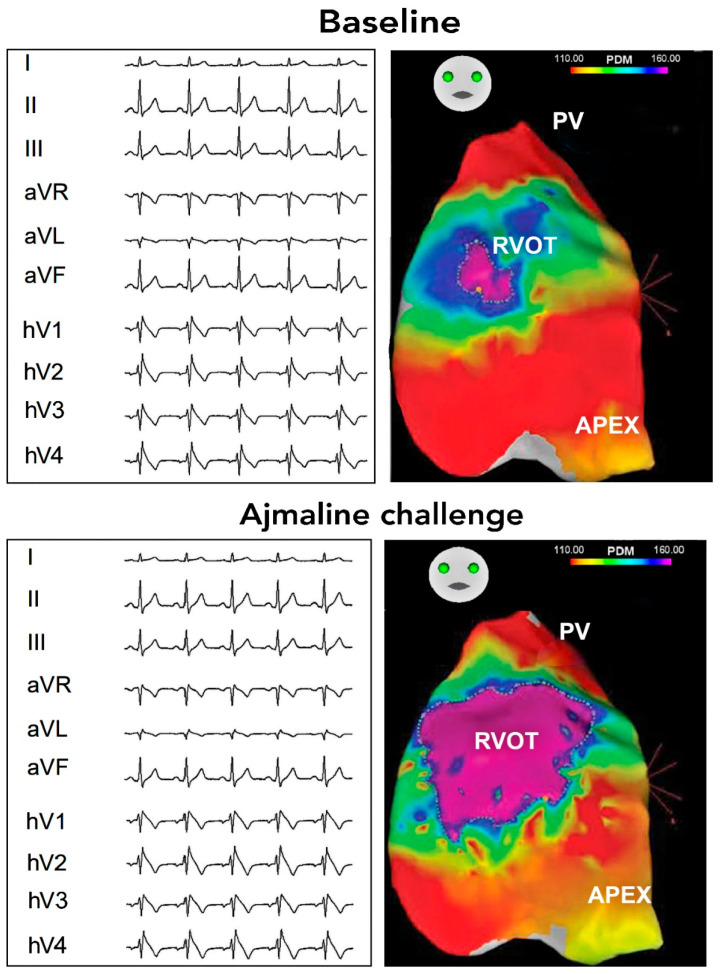
Epicardial electroanatomic mapping of a patient with Brugada syndrome, comparing baseline conditions with the effects of ajmaline administration. On the left, the surface ECG leads (I, II, III, aVR, aVL, aVF, hV1–hV4) demonstrate the transformation induced by ajmaline, with the emergence of a coved-type ST-segment elevation. On the right, the electroanatomic maps display the distribution of epicardial potentials, color-coded by bipolar voltage amplitude (PDM range: 110–160). Under baseline conditions, a localized region of fractionated, low-voltage electrograms is observed near the right ventricular outflow tract (RVOT). Following the ajmaline challenge, this area expands significantly, with a prominent enlargement of the low-voltage zone, corresponding to the arrhythmogenic substrate, extending to the pulmonary valve (PV).

Primary mechanisms underlying BrS pathophysiology have been suggested: depolarization delay and altered repolarization [24,25,26]. The depolarization hypothesis suggests that delayed activation in the RVOT compared to the rest of the right ventricle creates a voltage gradient that manifests as ST-segment elevation. Evidence from mapping studies indicates that significant conduction delays in the RVOT are common in BrS patients. The alternative repolarization hypothesis arises from studies showing that a difference in action potential duration between the endocardial and epicardial layers of the RVOT generates a transmural repolarization gradient. These gradients are exaggerated in BrS, likely maintained by fibrotic changes that hinder normal electrotonic coupling, facilitating the persistence of these gradients [27]. Myocardial biopsy data support this theory, identifying fibrosis and reduced connexin expression, which weaken cellular coupling, promoting the characteristic Brugada ECG changes [28]. This disruption in cell coupling due to fibrotic changes exacerbates the endo-epicardial activation delay in the RVOT, reinforcing the ST-segment elevation on the ECG without necessarily relying on shortened epicardial action potentials as the primary mechanism. Histopathological analyses guided by electroanatomical mapping (EAM) have uncovered fibrosis and lymphomononuclear infiltrates in most BrS cases, with these abnormalities correlating with low-voltage EAM regions [29]. Fatty tissue, fibrosis, and lymphocytic infiltration were identified in 52–55% of BrS patients with inducible VF and no imaging abnormalities [30]. Epicardial mapping in BrS patients frequently reveals fractionated electrograms, which are indicative of abnormal conduction, lending further support to the depolarization delay hypothesis [29]. The fractionation seen in these signals might also be a result of phase 2 reentry within the epicardium, contributing to arrhythmogenicity. Ajmaline, a sodium channel blocker, is noted to increase signal fractionation in affected epicardial regions (Figure 2), a finding consistent with the depolarization delay hypothesis [31].

In addition, this area contains connective tissue, coronary vessels, mesenchymal cells, fibroblasts, inflammatory cells, and epicardial adipose tissue (EAT). EAT, in particular, is tightly associated with the subepicardial myocardium, affecting it through paracrine signaling and metabolic modulation. Furthermore, the EAT can release cytokines and adipokines that alter cardiac electrical properties. The subepicardial myocardium itself has unique electrophysiological characteristics, including a shorter action potential and higher density of the Ito current, making it prone to conduction delays and blocks, particularly in the RVOT, where there is a decreased conduction reserve due to reduced expression of connexins and SCN5A proteins [32].

## 5. Epicardial Substrate Mapping

The arrhythmogenic substrate in BrS is typically located on the epicardial RVOT and can be identified through endocardial mapping in only a small subset of patients. For instance, in a series by Talib et al., endocardial substrates were found in only 19% of BrS cases [33].

Developed initially for managing arrhythmogenic substrates in Chagas’ cardiomyopathy, the epicardial approach later became crucial for BrS treatment, particularly with the introduction of the subxiphoid access to the pericardium [34]. In a landmark study, Nademanee and colleagues identified clusters of abnormal, prolonged, low-voltage electrograms in the RVOT epicardium as the main electrophysiologic substrate for BrS. Ablation of these areas effectively normalized the Brugada ECG pattern and rendered most patients non-inducible for VF, suggesting that delayed depolarization in the RVOT may be the primary mechanism in BrS [35]. Subsequent studies validated this approach, confirming that ablation of these epicardial regions reduced arrhythmic events and eliminated the characteristic ECG abnormalities.

Building on this, Brugada and colleagues [36] used a drug-challenge protocol to unmask additional abnormal regions in the epicardium before performing ablation. By administering flecainide, they increased the area of low-voltage fractionation, providing a more extensive target for ablation. The procedural success was confirmed post-ablation through a rechallenge with the drug, which verified the absence of the type 1 Brugada ECG pattern and reduced VF inducibility.

Concerning quantifying the extent of the arrhythmogenic substrate, Pappone and colleagues [37] found that there may be a critical threshold for substrate area in predicting VF inducibility, recommending that patients with larger substrate areas be prioritized for ablation. This quantification aids in stratifying patients by arrhythmic risk, particularly for those who may not meet conventional clinical criteria for high-risk designation.

Catheter ablation for BrS appears to eliminate the type 1 ECG pattern by either removing the abnormally shortened action potential epicardial layer or creating a full transmural lesion that neutralizes the abnormal voltage gradients. In theory, ablating a substantial area of the delayed depolarization zones ensures that any conduction delay between the RVOT and the remainder of the right ventricle is effectively neutralized, thereby preventing the typical ST-segment elevation [2,38,39,40].

Despite its benefits, the epicardial carries some risks, including pericardial complications and, in rare instances, severe adverse reactions to ajmaline used to enhance the functional substrate visualization. However, in experienced centers, these risks are generally manageable, allowing for significant clinical benefit in BrS patients with recurrent VF who are refractory to medical management or ICD therapy.

The prognostic implications of VF inducibility during PVS in BrS are still controversial due to inconsistencies in PVS results, including differences in the number of extra stimuli, minimal coupling intervals, and stimulation sites [41]. This can lead to false-positive VF inductions and limit the reproducibility of PVS data [42]. The accuracy of PVS may be enhanced using endocardial unipolar EAM. In fact, a correlation exists between RVOT abnormalities detected through endocardial high-density unipolar EAM and VF inducibility during PVS [43]. These observations are confirmed by a larger number of inducible versus non-inducible patients, which reported significantly larger endocardial unipolar low-voltage areas [30]. The same results are reported by epicardial mapping studies with broader epicardial abnormalities, characterized by fragmented and prolonged-duration ventricular potentials, in patients with VF inducibility compared to those without arrhythmias [2]. Incorporating endocardial and epicardial mapping into risk stratification for BrS represents a significant step forward in identifying patients at higher risk for arrhythmic events. Some recent experiences evaluated RVOT abnormalities between patients with aborted ASCD and documented VF and asymptomatic individuals with spontaneous type-1 ECG patterns. High-density endocardial and epicardial mapping revealed that all symptomatic patients displayed abnormal electroanatomical maps, with endocardial unipolar low-voltage areas predominantly located in the anterior RVOT colocalized with epicardial bipolar low-voltage areas. The extent of these abnormalities was significantly greater in symptomatic versus asymptomatic patients [44]. A proposed workflow for electrophysiological risk stratification in asymptomatic BrS patients emphasizes identifying abnormal RVOT substrates through mapping and assessing VF inducibility. Patients with both abnormal electroanatomical substrates and induced VF appear to be at heightened risk for future arrhythmic events

## 6. Substrate Ablation and Outcomes

Epicardial radiofrequency (RF) ablation has proven to be an effective strategy and has now achieved a certain consensus, showing the reproducibility of outcomes in different experienced centers (Table 2).

The procedure of RVOT ablation was first described in 2011, when ablation of the anterior RVOT epicardium rendered arrhythmias non-inducible during testing, normalized ECG patterns, and led to favorable outcomes at a mean follow-up of 20 months [35]. After ablation, patients revealed complete elimination of BrS-associated ECG changes and no VF recurrences over a median follow-up of three years. The stepwise approach began with targeting VF triggers, followed by endocardial mapping and ablation of abnormal ventricular electrograms characterized by low voltage, fractionation, or isolated late potentials. Epicardial substrate localization seems to improve from the infusion of sodium channel blockers administration to identify pathological areas. In fact, Pappone et al. reported normalization of the ECG in all 135 patients following epicardial ablation, with ajmaline rechallenge identifying additional areas requiring treatment in over 60% of cases [37], supporting the depolarization hypothesis. A novel hybrid approach involving thoracoscopic epicardial ablation has recently been described [49]. This technique combines direct visualization of the RVOT epicardial wall with EAM, allowing precise identification and ablation of pathological areas. Real-time monitoring during RF application facilitates higher power settings and shorter ablation times, producing more homogeneous lesions while minimizing risks such as coronary artery injury or steam pops. Despite these advantages, VA recurrences were observed in a subset of patients treated for secondary prevention. Complication rates for BrS ablation are generally low but vary by approach. Mild pericarditis in up to one-third of patients and more threatening acute hemopericardium in 2% of cases are reported [50]. Thoracoscopic techniques have demonstrated similar safety profiles, with complications such as pneumothorax and late hemopericardium occurring infrequently. In contrast, other centers report lower complication rates [37].

## 7. Discussion

Epicardial ablation represents a promising advancement in the treatment of BrS, particularly for addressing arrhythmogenic substrates that are inaccessible with endocardial techniques. However, given the current state of evidence, it seems prudent to reserve this approach for patients with recurrent ventricular arrhythmias who are already ICD carriers. These patients represent a population with a clear need for adjunctive therapies, as ICD shocks alone do not modify the underlying substrate and can significantly impact the quality of life. By targeting the epicardial substrate in these high-risk individuals, ablation may reduce arrhythmia recurrence, mitigate the burden of ICD interventions, and improve overall patient outcomes.

The epicardial approach to substrate ablation offers several advantages over traditional endocardial techniques. Thoracoscopic access, in particular, offers direct visualization of the RVOT and adjacent structures, such as the coronary arteries, but also enables more precise management of epicardial fat, which can otherwise hinder effective energy delivery. Direct visualization allows operators to achieve better catheter-tissue contact, facilitating higher-power RF delivery. This results in shorter RF application times and the creation of wider, more homogenous lesions, which are critical for ensuring long-term procedural success. Despite its advantages, epicardial ablation is not without risks. Its invasive nature and associated risks underscore the need for careful patient selection, ideally within specialized centers equipped to manage potential complications. Additionally, the administration of sodium channel blockers carries a potential risk of inducing malignant arrhythmias. Therefore, this step must be preceded by thorough patient counseling and conducted under controlled conditions. Another area of ongoing debate concerns the optimal use of sodium channel blockers during ablation. While these agents have been shown to enhance the identification of pathological areas, questions remain about which specific drug provides the most reliable substrate delineation and whether targeting these areas significantly improves procedural outcomes. Comparative studies exploring different pharmacological agents and their impact on substrate mapping could provide valuable insights to guide clinical practice. One of the most compelling aspects of RF ablation in BrS is its potential to reduce or even eliminate the need for ICD therapy in certain patients, particularly younger patients who require long-term device therapy. However, the evidence supporting the durability of ablation outcomes in BrS is still evolving, and long-term follow-up studies are needed to validate its efficacy as a standalone therapy. Advancements in imaging and mapping technologies are likely to further enhance the efficacy and safety of epicardial ablation in BrS. Techniques such as high-density mapping, integration of cardiac MRI data, and the use of novel energy sources (e.g., pulsed-field ablation) hold promise for more precise and less invasive treatments. Moreover, a deeper understanding of the genetic and molecular mechanisms underlying BrS could pave the way for personalized approaches to substrate modification tailored to the unique electrophysiological profile of each patient. In parallel, ongoing research into the role of sodium channel blockers and other pharmacological agents during ablation may help refine procedural protocols and reduce the need for repeat procedures.

Recent developments in artificial intelligence (AI) are reshaping our approach to the detection and risk stratification of BrS, with potential implications for guiding therapeutic interventions such as epicardial ablation. AI methods address inherent challenges in BrS diagnostics, particularly the transient nature of type 1 ECG patterns, which often appear only under provocation (e.g., sodium channel blockade or febrile states) [51]. Deep learning models, such as convolutional and recurrent neural networks, have demonstrated robust performance, with high discrimination metrics (AUC > 0.9) in both training and validation settings [52]. Notably, these models extend beyond static detection, enabling dynamic predictions of drug-induced type 1 ECG patterns and refining diagnostic accuracy in patients with ambiguous presentations. In parallel, AI-driven risk stratification has provided new insights into ventricular arrhythmia prediction and benefits from ICD implantation, leveraging advanced techniques like non-negative matrix factorization to enhance signal processing [53]. However, while early studies highlight the potential of AI to augment traditional risk scores, challenges remain in translating these methods into clinical practice, particularly due to computational complexity and the need for real-time applicability to realize better the potential value of epicardial ablation vs ICD implantation. These findings represent a significant step toward precision medicine in BrS, offering avenues to improve patient selection and effectively tailor interventions such as epicardial ablation

## 8. Conclusions

Despite ongoing debate about the mechanisms underlying BrS, the RVOT epicardium remains a central focus for ablation strategies. Advances in substrate mapping and tailored ablation techniques have significantly improved outcomes, making epicardial approaches a valuable technique for reducing arrhythmia recurrence, particularly for the mitigation of the burden of ICD interventions.

## Figures and Tables

**Table 1:** Key genes responsible for substrate in ablation response in BrS: functions, loci, and clinical relevance. I_Na_ = inward sodium current; I_CaL_ = L-type calcium channel; K_Ito_ = outward potassium current channel.

Gene	Locus	Function	Relevance in BrS	Polygenic Interaction
SCN5A	3p22.2	Encodes the Nav1.5 sodium channel	Mutations reduce I_Na_ density, impacting substrate extent and clinical severity	Truncation variants cause haploinsufficiency with severe phenotypes; H558R mitigates severity by enhancing I_Na_
SCN10A	3p22.2	Associated with I_Na_ regulation	Indirectly influences arrhythmogenic substrate characteristics	Affects cardiac conduction through interaction with SCN5A; specific variants reduce SCN5A expression, slowing conduction
CACNA1C	12p13.33	Encodes the I_CaL_	Variants may affect arrhythmogenic substrate characteristics	Associated with reduced I_CaL_ and altered calcium signaling, influencing the repolarization phase in BrS
KCND3	1p13.2	Encodes the K_Ito_	Gain-of-function variants increase Ito, contributing to phase-1 repolarization and arrhythmogenesis	Increased Ito contributes to action potential shortening, exacerbating arrhythmic risk.
KCNE3	11q13.4	Regulates the K_Ito_	Gain-of-function variants increase Ito, similar to KCND3	Variants enhance Ito, promoting substrate instability and arrhythmic predisposition.
ZFHX3	16q22.2	Transcription factor	Emerging modifier influencing conduction properties	Variants associated with atrial fibrillation; potential indirect role in BrS by modifying conduction pathways.
HEY2	6q22.31	Transcriptional regulator involved in heart development	Emerging modifier that may affect ion channel regulation	Variants influence ventricular conduction and ECG features, with links to BrS phenotype.

**Table 2:** Studies and outcomes of ventricular arrhythmia ablation using an epicardial approach in patients with BrS. BrS = Brugada Syndrome; SCD = sudden cardiac death; ICD = implantable cardioverter defibrillator; VF = ventriculur fibrillation; VA = ventricular arrhythmias; RVOT = right ventricular outflow tract; EGM = intracardiac electrogram; ERS = early repolarization syndrome.

Study	Design	Patients and Characteristics	Substrate/Ablation Target	Mapping and Ablation Approach	VA Recurrence
Nademanee et al. 2011 [35]	Prospective single center	9 patients with BrS and a history of aborted SCD and ICD implanted	late depolarization abnormalities characterized by low-voltage fractionated late potentials and VF triggers	Endocardial and/or epicardial	11%
Brugada et al. 2015 [36]	Prospective single center	14 patients with BrS and symptoms attributable to VA or high vulnerability for VA induction at electrophysiology study	Low-voltage areas	Epicardial	none
Zhang et al. 2016 [38]	Prospective multicenter	11 patients with BrS without ICD implantation	Low voltage areas in RVOT identified based on EGM	Endocardial and/or epicardial	27%
Chung et al. 2017 [39]	Retrospective single center	15 patients with BrS and a history of aborted SCD or episodes of VA	Epicardial scar/low voltage zone	Epicardial	7%
Pappone et al. 2017 [37]	Prospective single center	135 symptomatic patients with BrS and ICD implanted	Abnormal, long-duration bipolar electrograms	Epicardial	1,5%
Shelke et al. 2017 [40]	Prospective single center	5 patients with BrS and recurrent ICD shocks for VA	Fractionated, split, and late potentials with low voltage	Endocardial and/or epicardial	25%
Nademanee et al. 2019 [45]	Retrospective multicenter	33 patients with ERS with Brugada pattern, and 7 patients with ERS without Brugada pattern with VF or cardiac/unknown syncope	Low voltage area, split or fractionated EGM	Endocardial and/or epicardial	9%
Kamakura et al. 2021 [46]	Prospective single center	16 patients with BrS and at least 1 episode of VA	EGMs with low amplitude, long duration, split/late potentials	Endocardial and/or epicardial	none
Mamiya et al. 2021 [47]	Retrospective single center	11 patients with BrS and aborted sudden cardiac arrest or VA on electrophysiological study	Low voltage areas, fractionated signals, and delayed potentials	Epicardial	27%
Haïssaguerre et al. 2022 [48]	Retrospective multicenter	17 patients with BrS presenting sudden death due to VA	Epicardial and endocardial low-voltage areas	Epicardial	35%

## Data Availability

No new data were created.
